# Feeding Tall Fescue Seed during Mid and Late Gestation Influences Subsequent Postnatal Growth, Puberty, and Carcass Quality of Offspring

**DOI:** 10.3390/ani10101859

**Published:** 2020-10-12

**Authors:** Maslyn A. Greene, Jessica L. Britt, J. Keith Bertrand, James L. Klotz, William Bridges, John G. Andrae, Susan K. Duckett

**Affiliations:** 1 Department of Animal and Veterinary Sciences, Clemson University, Clemson, SC 29634, USA; maslyng@clemson.edu (M.A.G.); jlbritt@clemson.edu (J.L.B.); jkbertr@clemson.edu (J.K.B.); 2 USDA-ARS, Forage Production Research Unit, Lexington, KY 40546, USA; James.klotz@usda.gov; 3 Department of Mathematical Sciences, Clemson University, Clemson, SC 29634, USA; wbrdgs@clemson.edu; 4 Department of Plant and Environmental Sciences, Clemson University, Clemson, SC 29634, USA; jandrae@clemson.edu

**Keywords:** sheep, tall fescue, ergot alkaloids, growth, puberty, carcass quality

## Abstract

**Simple Summary:**

Little is known about the exposure to ergot alkaloids, a class of mycotoxins, during fetal development on subsequent post-weaning growth, puberty, and carcass quality of the offspring. This study evaluated post-weaning growth, puberty attainment, and carcass quality in lambs that were exposed to endophyte-infected or endophyte-free tall fescue seed during different stages of gestation. Puberty was delayed in ewe lambs that were exposed to ergot alkaloids during late gestation. Ergot alkaloid exposure had minor effects on growth and carcass traits in wether lambs fed high concentrates but alterations in fat deposition and tenderness were observed. These results indicate that exposure to ergot alkaloids during gestation does alter subsequent post-weaning puberty attainment and body composition of the offspring.

**Abstract:**

Weaned lambs (*n* = 82), born to ewes fed endophyte-free (E−) or endophyte-infected (E+; 1.77 mg hd^−1^ d^−1^ ergovaline + ergovalinine) tall fescue seed from d 35 to 85 of gestation (MID) and/or d 86 of gestation to parturition (LATE), were used to examine how ergot alkaloid exposure during fetal development altered subsequent puberty attainment or carcass quality. Lambs were weaned at 75 d of age and separated by sex to assess puberty in ewe lambs (*n* = 39) and to evaluate growth, carcass and meat quality in wethers (*n* = 43). Data were analyzed with maternal fescue treatment, stage of gestation, and two-way interaction in the model. Age at puberty tended (*P* = 0.06) to be longer for ewe lambs born to dams fed E+ fescue during LATE gestation versus those fed E−. Post-weaning average daily gain tended to be higher (*P* = 0.07) for wether lambs born to dams fed E+ fescue seed during MID gestation compared to E−. Exposure to ergot alkaloids during fetal growth altered (*P* < 0.10) longissimus muscle weight and color, lipid deposition, fatty acid composition, and shear force values of semimembranosus muscle in wether lambs. These results indicate that exposure to ergot alkaloids in utero does alter subsequent post-weaning puberty attainment and body composition in offspring.

## 1. Introduction

Consumption of tall fescue (*Lolium arundinaceum* (Scheyreb.) Darbysh) results in fescue toxicosis, a syndrome associated with reduced bodyweight gains in cattle [1,2,3] and lambs [4,5]. Beef calves are reported to be 14% lighter in weight at weaning when cow-calf pairs graze endophyte-infected (E+) tall fescue pastures compared to endophyte-free (E−). Growing cattle grazing E+ tall fescue pastures have on average growth rates that are reduced by 50% compared to E− (summary of 13 studies) [6]. Parish et al. [3] reported 41% reduction in lamb growth rate when grazing E+ tall fescue pasture compared to E−. Duckett et al. [7] who reported the lack of compensatory growth in feedlot steers that previously grazed E+ tall fescue pastures during the stocker phase; in contrast, others [8,9] observed compensatory effects of steers in the feedlot after grazing on E+ tall fescue pastures. Calves typically receive a discount at sale if they show signs of fescue toxicosis [10,11]. Poor animal performance is attributed to an endophyte (*Epichloë coenophiala*; previously *Neotyphodium coenophialum*) found within the plant that produces ergot alkaloids, a class of mycotoxins [12].

Less is known about impact of fescue toxicosis on fetal programming and subsequent postnatal growth of the offspring. Maternal consumption of ergot alkaloids via endophyte-infected tall fescue pasture or tall fescue seed results in reduced birthweights in calves [13] and lambs [14,15]. Further research suggests that reductions in offspring birth weights are due to intrauterine growth restriction (IUGR) [16]. Greenwood et al. [17] suggested that restrictions in maternal nutrition from 80 d of gestation to parturition limits subsequent postnatal growth of the offspring. Symonds et al. [18] documented that maternal nutrient restriction at specific stages of gestation can alter offspring long-term. Subsequent postnatal growth of offspring exposed to ergot alkaloids in utero has not been examined. The objective of this study is to examine how ergot alkaloid exposure during mid- and/or-late gestation alters subsequent post-weaning growth, puberty attainment, and carcass quality in lambs.

## 2. Materials and Methods

All animal experimental procedures were reviewed and approved by the Clemson University Institutional Animal Care and Use Committee (AUP 2014-081). All animals were housed at the Clemson University Small Ruminant Facility.

### 2.1. Experimental Design

Weaned lambs (*n* = 82) born to Suffolk ewes (173% lambing rate) of different dopamine receptor D2 (*DRD2)* genotypes (A|A, A|G, or G|G; NC_040266.1:26511792-26512187) fed endophyte-free (E−) or endophyte-infected (E+; ergovaline/ergovalinine 1.77 mg hd^−1^ d^−1^) tall fescue seed during MID (d 35 to 85) and/or LATE (d86 to parturition) gestation were used in this study to evaluate effects of ergot alkaloid exposure during specific stages of fetal development on subsequent post-weaning growth, puberty attainment, and carcass quality [19]. Lambs were weaned at 75 d of age and separated by sex.

### 2.2. Blood Samples

Blood samples were obtained from wether lambs that were born to the subset of ewes monitored for insulin sensitivity in the companion paper [19] from birth to finished weight. Blood samples were also collected from all ewe lambs on a weekly basis during the post-weaning growing period to assess puberty development. All blood samples were collected by jugular venipuncture into serum or ethylenediaminetetraacetic acid (EDTA) collection tubes with the use of a vacutainer. Plasma tubes were immediately placed on ice and centrifuged at 537× *g* for 20 min at 4 °C to collect plasma, which was stored at −20 °C for later analysis. Serum tubes were allowed to clot and centrifuged at 537× *g* for 20 min at 4 °C to collect serum, which was stored at −20 °C for later analysis.

### 2.3. Ewe Lambs

At weaning, ewe lambs (*n* = 39) were maintained on non-fescue pasture and supplemented with High Energy Lamb Grower feed (Southern States, Richmond, VA) for a targeted weight gain of 114 g/d. Ewe lambs were weighed biweekly and FAMACHA^©^ scored [20] to manage parasite loads. Ewe lambs were dewormed using Prohibit^®^ (levamisole hydrochloride; AgriLabs, St. Joseph, MO, USA) oral drench when FAMACHA^©^ scores of 4 or 5 were recorded according to manufacturer’s directions. When outbreaks of haemonchosis occurred, lambs scoring a 3, 4 or 5 were dewormed and other anthelmintics (Cydectin^®^, moxidectin, Bayer Animal Health, Whippany, NJ, USA; Valbazen, albendazole, Zoetis Inc., Kalamazoo, MI, USA) were used in rotation. Plasma samples were collected weekly to evaluate progesterone concentrations using a progesterone ELISA kit (Cayman Chemical, Ann Arbor, MI, USA) to estimate puberty attainment. Plasma samples were purified using methylene chloride to minimize cross reactivity during progesterone analysis. The progesterone ELISA kit had an inter-assay and intra-assay variance of 3.66% and 7.90%, respectively. Progesterone recovery was tested by spiking a known amount of progesterone into samples prior to extraction and yielded a 92.4% recovery. A threshold concentration of 1.0 ng/mL progesterone in plasma has been established as the level indicative of puberty attainment in ewe lambs [21,22,23].

### 2.4. Wether Lambs

At weaning, wethers (*n* = 43) were allocated to pens and individually fed a concentrate diet, ad libitum, twice daily and intake was recorded. Wethers were given a two-week period at the start of feeding where hay was provided to facilitate the transition to a concentrate diet. All wethers received High Energy Lamb Grower (Southern States) for the growing period (weaning–40.82 kg) and High Energy Lamb Finisher during the finishing period (40.82 kg–56.70 kg or d 185 post-weaning).

At the end of the finishing period, wethers were fasted for 12 h prior to slaughter and live weight was obtained. Wethers were transported 15 km to the Clemson University Meat Laboratory for slaughter. Hide, head, and feet were removed from the carcass and weighed with the pelt. The brain was then removed from the skull and weighed. The carcass was eviscerated and each component weighed. Weights of all organs (heart, lungs, thymus, liver, kidneys, spleen, and pancreas) and fat depots (kidney, visceral, omental, and mesenteric fat) were obtained. A portion of the liver was aliquoted and frozen at −20 °C for proximate and fatty acid analysis. The digestive tract was weighed to obtain a gut fill and then separated (rumen, reticulum, omasum, abomasum, a 30.5 cm section of the jejunum, small intestine, and large intestine), stripped of continents, and weighed again. The proximal jejunum section was obtained according to Meyer et al. [24]. In summary, the section began 10 cm caudal to the junction of the mesenteric and ileocecal veins. The section was cut and 30.5 cm was measured and removed, stripped of any digestive contents, and weighed. A hot carcass weight was obtained prior to chilling.

The carcass was chilled at 4 °C for 24 h and then the chilled carcass weight obtained. Carcasses were ribbed at 12/13th rib and standard carcass measurements collected. Instrumental color measurements were recorded for L* (measures darkness to lightness; lower L* indicates a darker color), a* (measures redness; higher a* value indicates a redder color), and b* (measures yellowness; higher b* value indicates a more yellow color) using a Minolta chromameter (CR-310, Minolta Inc., Osaka, Japan) with a 50-mm-diameter measurement area, which was calibrated using the ceramic disk provided by the manufacturer. The illuminant was A with 10 °C standard observer and triplicate measures were collected for subcutaneous fat and longissimus muscle in each rib. Color readings were determined on the exposed LM at the anterior (13th rib) of the loin after a 15 min bloom time and subcutaneous (SQ) fat covering the anterior loin. Values were recorded from three locations of exposed lean and SQ fat to obtain a representative reading.

From the left side, the major muscles (longissimus, psoas major and minor, gluteus medius, biceps femoris, semitendinosus, semimembranosus, adductor, quadriceps femoris, and gracilis) from rack, loin and leg were individually excised, trimmed of subcutaneous/intermuscular fat and connective tissue, and weighed. Chops (2.54 cm thick) were obtained from the gluteus medius, longissimus, semimembranosus, and semitendinosus muscles, vacuum packaged and held for 6 d at 4 °C prior to freezing for subsequent Warner Bratzler shear force (WBS) analysis. A steak (2.54 cm thick) was also removed from longissimus and semitendinosus muscles, vacuum packaged and frozen at −20 °C for subsequent proximate and fatty acid analyses. From the right side of the carcass, all muscle and fat were removed from bone, ground and weighed. A sample of ground lamb was obtained for subsequent proximate and fatty acid analyses.

### 2.5. Proximate Composition

Ground lamb, collected from the right side of each carcass, was thoroughly mixed and multiple grab samples were obtained for proximate analysis. Ground lamb and individual muscle samples were individually chopped and mixed (Blixer3 Series D, Robot Coupe Inc., Ridgeland, MS, USA). An aliquot was taken for moisture content analysis by drying the samples for 24 h at 100 °C and calculating weight loss. The remaining sample was frozen at −20 °C overnight, lyophilized, (VirTis, SP Scientific, Warminster, PA), mixed again (Blixer3), and kept at −20 °C. Muscle samples, in duplicate, were evaluated for nitrogen content by the combustion method using a Leco FP-2000 N analyzer (Leco Corp., St. Joseph, MI, USA). Nitrogen amount was multiplied by 6.25 to calculate the crude protein level of the samples. Mineral content was measured by ashing the samples for 8 h at 600 °C. Total lipid content of the samples was measured in duplicate utilizing an Ankom XT-15 Extractor (Ankom Technology, Macedon, NY, USA), and hexane as a solvent.

### 2.6. Fatty Acid Analysis

Fatty acid analysis was conducted on the total lean tissue, liver, longissimus, and semitendinosus according to [14]. In brief, tissue samples were first lyophilized and transmethylated following the protocol of Park and Goins [25]. Fatty acid methyl esters (FAME) were assessed using a gas chromatograph (Agilent 6850, Agilent, San Fernando, CA, USA) equipped with an automatic sampler (Agilent 7673A, Hewlett-Packard, San Fernando, CA, USA). Fatty acid separations were accomplished with a 120-m TR-FAME (Thermo Fisher, Greenville, SC, USA) capillary column (0.25 mm i.d. and 0.20 μm film thickness). Column oven temperature increased from 150 °C to 160 °C at a rate of 1 °C/min, then from 160 °C to 167 °C at a rate of 0.2 °C/min, and then from 167 °C to 225 °C at a rate of 1.5 °C/min. Column temperature was maintained at 225 °C for 16 min. Injector and detector temperatures were maintained at 250 °C. Sample injection volume was 1 μL. Hydrogen was the carrier gas, at a flow rate of 1 mL/min. Each sample was run in duplicate for analysis, as well as at a split ratio of 10:1 and 100:1. Retention times of known standards (Sigma, St. Louis, MO, USA; Supelco, Bellefonte, PA, USA; Matreya, Pleasant Gap, PA, USA) were used to identify sample fatty acids. Quantification of fatty acids was done by adding an internal standard, methyl tricosanoic (C23:0), during methylation, and is expressed as a percentage of the total fatty acid weight.

### 2.7. Statistical Analysis

The univariate procedure of SAS (SAS 9.4, SAS Inst. Inc., Cary, NC, USA) was used to test all variables for normality. Lamb was considered the experimental unit for all data collected from post-weaning to finishing or puberty. Data were analyzed as a 3 × 2 × 2 factorial arrangement of maternal DRD2 genotype, fescue treatment, stage of gestation, and all interactions in the model using the Mixed procedure of SAS (SAS 9.4, SAS Inst. Inc., Cary, NC, USA). Maternal DRD2 genotype and all interactions with genotype were non-significant for all traits and removed from the final model. The final model was a 2 × 2 factorial with fescue treatment, stage of gestation and interaction. Least square means were generated and tested using protected least significance difference test. For blood metabolites and ewe lamb growth post-weaning, the model also included time and all interactions with time were tested. For proximate and fatty acid analyses, tissue was included in the model and all interactions with tissue were tested. Significance was determined at a probability ≤ 0.05 with trends at a probability ≤ 0.10. Breeding potential of ewe lambs was analyzed as a Chi-Square analysis using JMP software (SAS).

## 3. Results

### 3.1. Puberty Attainment

**Figure 1:**
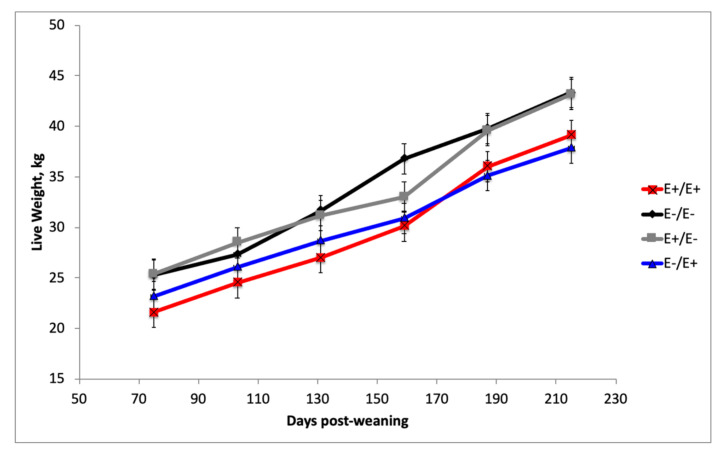
Live weight during post-weaning growth of ewe lambs born to ewes fed endophyte-free (E−) or endophyte-infected (E+) tall fescue seed during MID (d35 to 85) and/or LATE (d 85 to parturition) gestation.

Ewe lambs grazed non-fescue pastures and were supplemented to achieve a minimum average daily gain (ADG) post-weaning. There was an interaction (*p* = 0.04) between fescue seed treatment by stage of gestation over time during postweaning growth (Figure 1). Live weight of the ewe lambs was greater (*p* < 0.05) for ewe lambs born to ewes fed E− fescue during LATE gestation compared to E+ during postnatal growth (Figure 1). Age at puberty tended (*p* = 0.06) to be longer for ewe lambs born to dams fed E+ fescue during LATE gestation versus those fed E− (196.4 vs. 185.6 d; Table 1). Weight at puberty did not differ (*p* > 0.05) for ewe lambs by maternal fescue seed treatment. If we evaluate breeding potential at 6 mo of age, ewe lambs born to ewes fed E+ fescue seed during LATE gestation had lower (*p* < 0.05) percentages reaching age or both age and weight at the beginning of the breeding season (6 mo of age).

### 3.2. Wether Growth and Organ Weights

Wether lambs were individually fed a grower/finishing ration, ad libitum until they reached 55 kg body weight (BW) or 185 d of age (Table 2). Maternal fescue seed treatment did not alter (*p* > 0.20) total days on feed, final live weight, dry matter intake (DMI), gain:feed or endpoint (Table 2). Average daily gain tended to be higher (*p* = 0.07) for wethers born to dams fed E+ fescue seed during MID gestation compared to E−. At slaughter, all organs were removed and individually weighed. Digestive tract components were flushed with water to remove all digesta and weighed empty. No differences (*P* > 0.10) were observed in organ mass or viscera weight based on maternal exposure to fescue seed treatments except for a section of the proximal jejunum. The weight a 30.5 cm section of the proximal jejunum was lower *(P* = 0.01) for wethers born to ewes fed E+ fescue seed during MID gestation compared to wethers from ewes consuming E− fescue seed. This difference remained even when converted to a percentage of empty body weight basis.

### 3.3. Carcass Characteristics

Hot carcass weight and dressing percentage did not differ (*p* > 0.10) by maternal fescue seed treatment (Table 3). Chilled carcass weight and other carcass quality measures did not differ (*p* > 0. 10) by maternal fescue seed treatment except for flank streaking and quality grade. Flank streaking and quality grade were lower (*p* < 0.05) in carcasses from wethers born to dams fed E+ fescue seed during LATE gestation. Redness (a*) and yellowness (b*) objective color measures of the longissimus muscle and subcutaneous fat did not differ (*p* > 0.10) by maternal fescue treatment. Lightness (L*) values of the subcutaneous fat were highest (*p* = 0.02) in wether carcasses from E+/E+ dams and lowest (*p* = 0.02) for E+/E− wether carcasses. Lightness values of longissimus muscle were greater (*p* = 0.04) for wether carcasses from dams fed E+ fescue during LATE gestation compared to MID gestation.

### 3.4. Fat Depots and Tenderness

The interaction between fescue treatment and stage of gestation was significant for kidney fat amount. Wethers born to E+/E+ ewes tended to have greater kidney fat amounts compared to other fescue treatments (*p* = 0.09; Table 4). Visceral fat mass also tended (*p* = 0.05) to be greater in wethers born to ewes fed E+ fescue seed during LATE gestation. Weight of the longissimus muscles was greater (*p* = 0.031) for wethers born to ewes consuming E+ fescue seed during MID gestation compared to E− fescue seed. Longissimus weight, as a percentage of the empty body weight, tended (*p* = 0.06) to be increased for wethers from E+/E− ewes compared to all other treatments. All other muscles excised did not differ (*p* > 0.10) by maternal fescue exposure. Carcass composition was not altered (*p* > 0.15) by maternal fescue treatment as evaluated physical dissection and chemical analyses. Longissimus, gluteus medius, and semitendinosus muscle shear force values did not differ by fescue treatment. The interaction for fescue treatment and stage of gestation was significant for shear force in the semimembranosus muscle. Shear force values were higher (*p* < 0.05) for wethers born to E−/E+ ewes compared to other fescue treatments.

### 3.5. Total Lipid Content

Several tissues (ground lamb, liver, longissimus muscle and semitendinosus muscle) were analyzed for proximate composition and long chain fatty acid analyses to ascertain any changes at the tissue level (Table 5). Proximate composition (moisture, protein and total lipid) of the various tissues did not differ (*p* > 0.05) among maternal fescue seed treatment. Lipid and moisture content did differ (*p* < 0.01) by tissue type with ground lamb having the highest total lipid content and lowest moisture content compared to other tissues. Liver tissue had the lowest total lipid and the muscles had that highest moisture content.

### 3.6. Fatty Acid Composition

Long-chain fatty acid composition of four different tissues, ground lamb (GL), liver, longissimus muscle (LM) and semitendinosus muscle (ST) was altered by maternal fescue seed treatments (Table 6). Two and three-way interactions between fescue seed treatment during MID or LATE gestation and tissue were not significant except for C20:0, C20:1, C20:4 and C20:5, which are shown in graphs. Fatty acid analysis by fescue seed treatment averaged over tissue type is shown in Table 6. Myritic (C14:0), pentadecylic (C15:0), and total saturated fatty acid concentrations were greater (*p* = 0.03 and 0.01) in tissues from wethers born to dams fed E+ fescue during LATE gestation compared to those fed E−. Palmitic (C16:0) and trans-11 vaccenic (C18:1 trans-11) acid concentration tended (*p* = 0.09 and 0.08) to be greater in tissues from wethers born to dams fed E+ fescue during LATE gestation. The two-way interaction between fescue seed treatment and stage of gestation was significant (*p* < 0.05) for trans-10 octadecenoic, linoleic, total monounsaturated (MUFA) and ratio of n-6 to n-3 fatty acids. For trans-10 octadecenoic (C18:1 trans-10), linoleic (C18:2), and n-6 to n-3 ratio, concentrations were greater (*p* < 0.05) in tissues from E−/E− and E+/E+ compared to E+/E− and E−/E+. Margaric (C17:0), an odd-chain fatty acid, concentration was greater (*p* = 0.01) for E−/E+ treatment compared to all other treatments. Stearic (C18:0) acid concentration was lower (*p* = 0.01) in all tissues for wethers born to E+/E+ ewes compared all other treatments. Concentrations of cis-11 vaccenic acid tended to be greater (*p* = 0.08) for E−/E− compared to E−/E+. Total monounsaturated fatty acid concentration was highest (*p* = 0.01) for E+/E− and lowest (*p* = 0.01) for E+/E+ treatments.

**Figure 2:**
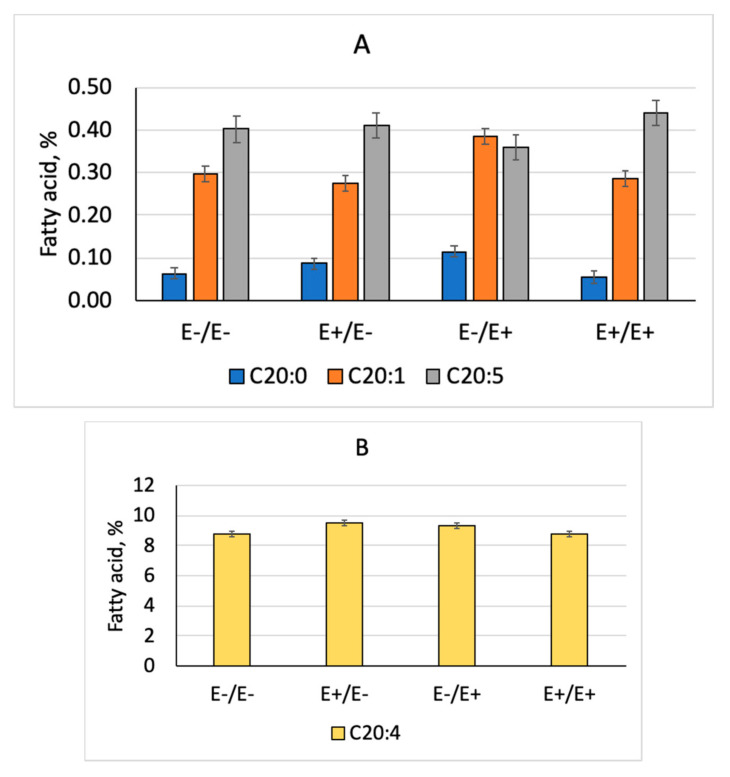
Long-chain 20C fatty acid concentrations in livers of wethers born to ewes consuming endophyte-infected (E+) or endophyte-free (E−) fescue during MID (d 35-85) and LATE (d 86- parturition) gestation. The interaction between fescue seed treatment and stage of gestation was significant (*p* < 0.05).

The three-way interactions between tissue type, fescue seed treatment and stage of gestation was significant for arachidic (C20:0, *p* = 0.022), eicosadienoic (C20:2, *p* = 0.032), arachidonic (C20:4, *p* = 0.048), and eicosapentaenoic (EPA, C20:5, *p* = 0.05). The changes in fatty acid concentrations with fescue seed treatment by stage of gestation were all observed in the liver tissue only. Arachidic acid concentration was greater for E+/E− and E−/E+ compared to E−/E− and E+/E+ in the liver (Figure 2A). Eicosadienoic acid concentration was greatest for E−/E+ fescue treatment compared to other fescue treatments in the liver (Figure 2A). Arachidonic acid (C20:4) concentration was greater for E+/E− and E−/E+ compared to E−/E− and E+/E+ in the liver (Figure 2B). For EPA, concentration was lowest for E−/E+ in the liver only (Figure 2A). Fatty acid composition differed (*p* < 0.05) by tissue type (data not shown) but changes due to maternal fescue treatment were consistent across all tissue types except for 20-C fatty acids where changes only occurred in the liver.

### 3.7. Serum Metabolites

We examined changes in glucose, insulin, and non-esterified fatty acids (NEFA) during the postnatal growth period to examine insulin sensitivity in wethers born to ewes fed E+ or E− fescue seed during gestation. Glucose, insulin, and NEFA concentrations or glucose to insulin ratio or revised quantitative insulin sensitivity check index (RQUICKI ) values did not differ (*p* > 0.28) due to fescue seed treatment or the interaction with sampling time; however, these values did change across the growth period (Table 7). Glucose concentrations were lower (*p* = 0.01) at birth compared to weaning, end of grower phase or finishing phase. Insulin concentrations were highest (*p* = 0.02) at the end of the grower phase and lowest at birth. This resulted in the glucose to insulin ratio being lower (*p* = 0.01) at end of the grower phase, indicating greater insulin sensitivity. Non-esterified fatty acid concentrations tended (*p* = 0.09) to be highest at weaning and lowest at end of the finishing phase. Insulin sensitivity, as measured using RQUICKI calculation, did not differ (*p* > 0.28) by treatment, time or the two-way interaction.

## 4. Discussion

This study was designed to examine how feeding tall fescue seed containing ergot alkaloids to ewes during mid and/or late gestation altered subsequent post-weaning growth, carcass quality, and puberty attainment of the offspring. After weaning, ewe lambs and wethers were raised under different systems that represent the typical production systems for each sex. Ewe lambs born to ewes fed E+ fescue seed during LATE gestation were lighter in weight from weaning to 215 d of age. Weight at puberty did not differ by maternal fescue seed treatments; however, age at puberty was delayed for ewe lambs exposed to E+ fescue seed treatment during LATE gestation. If we evaluate breeding potential for the fall breeding season at 6 mo of age, ewe lambs born to dams that were fed E+ fescue seed during LATE gestation had lower percentages of ewe lambs that were pubertal at 6 mo of age. Similarly, others also observed lower percentages of ewes reaching puberty [26] and reduced reproductive rates [27] in yearling ewes grazing E+ tall fescue pastures. In cattle, research has shown lower serum progesterone concentrations [28,29,30,31] and fewer heifers reaching puberty at the start of the breeding season (15 mo of age) when grazing E+ fescue pastures. Ross and co-workers [32] suggested feeding E+ fescue seed to mice appears to have a greater impact on reproduction in female than it does in the male. In this study, ewe lambs born to dams fed E+ seed during LATE gestation failed to reach puberty by 6 mo of age, when the breeding season would begin and therefore more aggressive development feeding program would be needed for these lambs to accelerate growth and hasten puberty.

Average daily gain of the wethers finished on high concentrates, ad libitum, was greater for lambs born to dams fed E+ seed during MID gestation. However, no differences in days on feed, intake, feed efficiency or endpoint were observed. The lambs exposed to E+ fescue seed during MID gestation had lower preweaning growth rates from birth to d 56 and the ewes had reduced milk production at d 2 and 21 of lactation [19]. These lower growth rates may have resulted in compensatory growth that allowed these lambs to catch-up with their contemporaries. Louey et al. [33] proposed that low birth weight lambs could catch-up body weight during early postnatal growth but that adiposity is increased. In cattle, others [8,9] have reported compensatory gains of cattle that grazed E+ fescue pastures prior to feedlot entry; however, Duckett et al. [7] found steers that grazed E+ fescue compared to E− or novel fescue pastures prior to feedlot entry were lighter at arrival and remained lighter throughout the 112 d finishing period.

Concentrations of serum or plasma metabolites in wethers across the growth curve did not show any changes due to ergot alkaloid exposure under normal conditions. Previous research indicated that lambs born to ewes fed E+ fescue during gestation may have altered insulin sensitivity which was associated with changes in muscle fiber miRNA expression [16]. Others [34] have observed changes in insulin sensitivity of offspring born to ewes that were nutrient restricted during late gestation (d 110 to 145) but that this depended on the rate of growth during the pre-weaning and post-weaning periods. They found that accelerated pre-weaning growth and greater obesity during post-weaning both increased insulin resistance and expression of genes associated with energy sensing. The apparent compensatory growth in offspring exposed to ergot alkaloids during MID gestation may be related to lack of change in insulin sensitivity. It is important to note that our glucose and insulin measures were collected under normal conditions and not during a glucose tolerance test as utilized by Dellschaft et al. [34] which may also have influenced results.

Exposure to ergot alkaloids in utero did not alter organ or digestive tracts weights, except for a reduction in the weight of the proximal jejunum. Lambs born to dams fed E+ seed during MID gestation had lower weights of the proximal jejunum section. Reductions in weight are generally associated with reductions in villus and crypt density, which suggest a reduction in functional area in the small intestine [35]. Wang and co-workers [36] also noted reductions in small intestine weights of IUGR piglets that were associated with reduced mucosal mass, malabsorption of nutrients, proteolytic activity and oxidative stress.

Differences in muscle weight at finishing were only observed for the longissimus muscle. Wethers from dams fed E+ seed during MID gestation had larger longissimus muscle mass at market weights compared to those fed E−. In contrast, our earlier research showed reductions in hind limb muscle mass when fetuses were exposed to E+ fescue seed during LATE gestation when examined at d 133 of gestation [16]. Muscle fiber number is set before birth and hyperplasia is reported to be complete by 85 d of gestation in the sheep [37]. Exposure to ergot alkaloids during MID gestation may have altered secondary myogenesis to reduce secondary muscle fiber number in those lambs. Others have shown that longissimus muscle of runt pigs [38] and semitendinosus muscle in lambs born to ewes under-fed during mid gestation [39,40] remain different during postnatal growth. Late gestational undernutrition has been known to cause alterations in muscle growth that can be seen at maturity [41].

Carcass characteristics and composition of the wether lambs was also not altered with maternal fescue seed treatment except for fat depots. Lambs born to dams fed E+ seed during both MID and LATE gestation had greater kidney fat depots. Lambs born to dams fed E+ fescue seed during LATE gestation also had greater visceral fat depots. Flank streaking, a measure of intramuscular fat deposition in lambs, and quality grade were lower in carcasses from lambs exposed to E+ fescue seed during LATE gestation. Increased adiposity of kidney and pelvic region in male offspring has been observed after maternal undernutrition during early to mid-gestation in sheep [42]. Greenwood and Bell [43] also found that low birthweight, IUGR lambs had greater adiposity at any given weight during postnatal growth. Exposure to ergot alkaloids during fetal development altered fat deposition in the lambs at market weight, which is similar to reports for IUGR lambs that accumulate greater lipid mass during the finishing period [18,42,43].

Due to the observed changes in deposition of fat in the lambs exposed to ergot alkaloids, we examined fatty acid compositional differences in several tissues (liver, longissimus, semimembranosus, and ground carcass) to examine how fatty acid synthesis may be altered with exposure to ergot alkaloids in utero. Exposure to ergot alkaloids during MID and LATE gestation altered tissue fatty acid composition with changes in saturated and eicosanoid fatty acid concentrations. Others have reported similar changes in stearic acid and eicosanoids in fetal muscle of lambs born to ewes fed E+ tall fescue [14] and total saturated and monounsaturated fatty acid content of beef from steers finished on E+ tall fescue pastures before slaughter [44]. Rumsey et al. [45] discovered greater stearic and lower oleic acid concentrations in necrotic fat samples from cows grazing fertilized E+ tall fescue pastures. Ergopeptine ergot alkaloids are absorbed and transported via the lymphatic system [46], similar to dietary lipids, which may explain alterations in lipid composition of tissues from animals exposed to E+ fescue. Unsaturated fatty acids of 20C chain lengths are classified as eicosanoids, which are biologically active compounds involved in prostaglandin, thromboxane, leukotriene, and lipotoxin biosynthesis [47]. Alterations in concentrations of these eicosanoids with exposure to ergot alkaloids in utero suggest that these fatty acids may be of importance for the response to mycotoxins and need further investigation.

Lightness values for subcutaneous and longissimus muscle color measurements were altered with maternal fescue treatment, whereas redness and yellowness were not affected. Others found that finishing steers on E+ tall fescue pastures [44] or feeding tall fescue seed [48] did not alter lean or subcutaneous fat color measures. In contrast, Baublits et al. [49] reported darker, more yellow color of the longissimus muscle of steers grazing E+ tall fescue compared to those grazing E+ tall fescue and supplemented with soyhulls. In this study, we observed increased shear force values in the semimembranosus muscle of lambs that were exposed to E+ fescue only during LATE gestation. Shear force values of other muscles (longissimus, gluteus, and semitendinosus) were not affected in this study. Others have shown that feeding tall fescue seed during the stocker phase [48] or finishing cattle on E+ fescue pastures [44,50] did not impact shear force values in longissimus muscle. Underwood et al. [51] reported higher shear force values and lower total lipid content of longissimus muscle in steers born to dams that were under-fed during mid-gestation. The semimembranosus muscle is the largest muscle in the lamb leg, which is the highest value cut of the lamb carcasses. Reductions in tenderness of the semimembranosus muscle with ergot alkaloid exposure during LATE gestation could reduce consumer acceptability of high-value lamb cuts.

## 5. Conclusions

Offspring born to dams that were fed endophyte-infected tall fescue seed during MID and LATE gestation had altered post-weaning growth rates, puberty, fat deposition and composition, and tenderness. Ewe lambs took longer to reach puberty and would not be ready for the breeding season at 6 mo of age. Wethers appeared to have compensatory growth when finished on concentrates with few changes in organ, digestive tract, or muscle weights; however, they had greater fat deposition, altered fatty acid composition and tougher leg muscles when exposed to ergot alkaloids in utero. These results demonstrate that exposure to ergot alkaloids in utero alters subsequent post-weaning performance, body composition and meat quality.

## Figures and Tables

**Table 1:** Post-weaning growth rates in ewe lambs born to ewes fed endophyte-infected (E+) or endophyte-free (E−) tall fescue seed from d 35 to 85 of gestation (MID) or d 86 of gestation to parturition (LATE) ^1^.

	Maternal Fescue Seed Treatment ^1^				
Ewe Lambs	E−/E−	E+/E−	E−/E+	E+/E+	Standard Error (SE)
No. born	7	11	12	9	
No. raised to wean	7	11	11	9	
No. raised to puberty, *n*	5	10	9	7	
**Puberty**					
Age ^2^, d	188 ^d^	183^d^	194^c^	199 ^c^	13.20
Weight ^2^, kg	42.86	40.10	36.63	38.84	7.15
**Breeding Potential**					
6 mo of age, %	38 ^a^	48 ^a^	17 ^b^	0 ^b^	
60% of mature wt ^3^, %	20	36	19	15	
Both age and weight, %	18 ^a^	44 ^a^	0^b^	0 ^b^	

^1^ Fescue seed treatment: ewe lambs born to ewes were fed endophyte-free (E−) or endophyte-infected (E+) tall fescue seed during MID gestation (d35 to 85) and/or LATE gestation (d 86 to parturition). ^2^ Age or weight at which plasma progesterone concentration exceeded threshold of 1.0 ng/mL, which is indicative of puberty attainment in ewe lambs. ^3^ Breeding potential was based on reaching puberty at 60% of mature weight (40.9 kg weight threshold for 68.2 kg mature weight). ^a,b^ Means in the same row with uncommon superscripts differ (*p* < 0.05). ^c,d^ Means in the same row with uncommon superscripts differ (*p* < 0.10).

**Table 2:** Post-weaning growth rate, final weights, organ and digestive tract weight of wethers born to ewes consuming endophyte-infected (E+) or endophyte-free (E−) fescue during MID (d 35-85) and/or LATE (d 86-parturition) gestation.

	Maternal Fescue Treatment ^1^				
	E−/E−	E+/E−	E−/E+	E+/E+	SE
Lambs Weaned	11	10	11	11	
Lambs Finished^2^	11	10	10	11	
**Performance**					
Days on feed, d	171.4	161.1	161.0	160.6	17.34
Dry matter intake (DMI), kg/d	1.09	1.12	1.15	1.15	0.123
Average daily gain (ADG), g/d	164.3 ^d^	176.8 ^c^	165.2 ^d^	178.4 ^c^	22.78
Gain:feed	0.15	0.16	0.15	0.16	0.020
Endpoint ^2^	1.67	1.49	1.65	1.74	0.474
**Weights**					
Final shrunk body weight (BW), kg	53.03	53.59	52.75	53.71	4.583
Empty BW, kg	44.30	44.58	44.32	45.70	3.572
Pelt, kg	9.12	9.13	8.82	9.54	0.854
Gut fill, kg	8.73	9.01	9.37	88.10	1.238
**Organs**					
Heart, g	258.8	276.9	280.6	242.9	66.05
Lungs, g	567.7	643.7	632.0	652.7	210.8
Kidneys, g	122.8	122.6	114.9	116.6	16.14
Liver, g	733.0	723.8	707.7	746.9	92.15
Spleen, g	86.6	90.4	77.5	95.0	26.70
Pancreas, g	70.3	72.8	71.2	67.9	12.32
Thymus, g	48.4	48.4	64.8	64.3	42.64
Brain, g	104.4	99.0	102.0	100.1	9.28
**Digestive Tract**					
Rumen, g	861.3	879.9	822.9	886.2	139.4
Reticulum, g	101.2	104.2	101.2	100.6	18.62
Omasum, g	115.9	106.1	111.4	103.9	33.92
Abomasum, g	214.3	230.3	221.3	206.6	53.10
Small intestine, g	596.8	686.7	624.6	683.9	199.1
Proximal jejunum ^3^, g	18.18 ^a^	13.47 ^b^	16.12 ^a^	14.33 ^b^	3.972
Large intestine, g	582.4	610.5	612.5	620.2	111.6

^1^ Fescue seed treatment: ewe lambs born to ewes were fed endophyte-free (E−) or endophyte-infected (E+) tall fescue seed during MID gestation (d35 to 85) and/or LATE gestation (d 86 to parturition). ^2^ Endpoint code: 1 = weight (56.70 kg), 2 = days (185 d postweaning). One wether (E−/E+) broke his rear leg and was euthanized during the finishing study. ^3^ Proximal jejunum: 30.5 cm section of the jejunum was removed cleaned and weighed. ^a,b^ Means in the same row with uncommon superscripts differ (*p* < 0.05). ^c,d^ Means in the same row with uncommon superscripts differ (*p* < 0.10).

**Table 3:** Post-weaning carcass composition and color measurements of wethers born to ewes consuming endophyte-infected (E+) or endophyte-free (E−) fescue during MID (d 35-85) and/or LATE (d 86- parturition) gestation.

	Maternal Fescue Treatment ^1^				
	E−/E−	E+/E−	E−/E+	E+/E+	SE
**Carcass Characteristics**					
Hot carcass weight, kg	28.79	28.12	27.36	28.59	2.441
Dressing percent, %	53.22	53.38	52.14	53.48	1.712
Cold carcass wt., kg	27.81	28.34	27.21	28.15	2.402
Cooler shrink, %	1.31	1.15	1.05	2.09	1.354
Ribeye area, cm^2^	18.12	19.20	18.10	17.86	2.100
Fat thickness, cm	0.40	0.41	0.47	0.45	0.170
Body wall depth, cm	1.82	1.63	1.76	1.82	0.405
Flank streaking ^2^	18.45 ^a^	18.30 ^a^	17.60 ^b^	18.00 ^b^	0.907
Conformation ^2^	18.0	18.6	17.7	18.0	1.044
Quality grade ^2^	18.4 ^a^	18.3 ^a^	17.7 ^b^	17.9 ^b^	0.907
Yield grade	2.05	1.78	2.25	2.22	0.672
**Carcass Composition**					
Fat-free Lean, %	57.00	57.04	56.65	55.51	2.57
Bone, %	21.68	21.32	21.53	21.72	2.51
Total Lipid, %	21.32	21.64	21.82	22.78	2.06
**Color Measurements**					
Subcutaneous fat					
Lightness (L*)	77.34 ^b^	74.26 ^c^	76.28 ^b^	79.23 ^a^	4.180
Redness (a*)	14.76	16.45	15.64	12.07	5.463
Yellowness (b*)	12.15	12.16	11.96	10.94	2.418
Longissimus muscle					
L*	42.15 ^b^	43.30 ^b^	43.49 ^a^	44.42 ^a^	1.836
a*	20.60	21.62	21.27	21.39	2.515
b*	5.94	6.27	6.23	6.53	1.026

^1^ Fescue seed treatment: wethers born to ewes were fed endophyte-free (E−) or endophyte-infected (E+) tall fescue seed during MID gestation (d35 to 85) and/or LATE gestation (d 86 to parturition). ^2^ Numerical code: 16 = Good^+^, 17 = Choice^−^, 18 = Choice^o^, 19 = Choice^+^, 20 = Prime^−^. ^a,b^ Means in the same row with uncommon superscripts differ (*p* < 0.05). ^c^ Means in the same row with uncommon superscripts differ (*p* < 0.10).

**Table 4:** Post weaning adipose and muscle weights, and Warner–Bratzler shear force values in carcasses from wethers born to ewes consuming endophyte-infected (E+) or endophyte-free (E−) fescue during MID (d 35-85) and LATE (d 86- parturition) gestation.

	Maternal Fescue Treatment ^1^				
	E−/E−	E+/E−	E−/E+	E+/E+	SE
**Fat Mass, g**					
Kidney fat	442.7 ^d^	393.2 ^d^	402.1 ^d^	485.8 ^c^	127.5
Visceral fat	1178.4 ^d^	1010.7 ^d^	1276.3 ^c^	1286.4^c^	288
**Individual Muscles, g**					
Longissimus	1621.4 ^b^	1782.1 ^a^	1585.4 ^b^	1669.9 ^a^	183.42
Gluteus medius	625.9	636.5	604.4	583.9	86.70
Semitendinosus	359.5	355.3	331.6	349.7	43.24
Psoas major/minor	311.4	339.2	291.4	318.9	77.00
Biceps femoris	898.7	900.6	820.5	900.5	131.76
Adductor	353.0	374.2	342.4	358.9	48.56
Semimembranosus	900.7	920.2	839.5	897.0	127.76
Quadriceps femoris	1217.5	1205.0	1145.3	1167.0	133.14
Gracilis	176.4	194.8	156.0	181.5	57.76
Total muscle	6464.4	6707.9	6116.4	6427.3	178.66
**Warner-Bratzler Shear Force, kg**					
Longissimus muscle	1.55	1.74	1.63	1.72	0.568
Gluteus Medius	1.42	1.44	1.63	1.44	0.301
Semimembranosus	2.04 ^b^	2.32 ^b^	2.61 ^a^	2.24 ^b^	0.084
Semitendinosus	1.92	1.94	2.10	1.75	0.381

^1^ Fescue seed treatment: wethers born to ewes fed endophyte-free (E−) or endophyte-infected (E+) tall fescue seed during MID gestation (d35 to 85) and/or LATE gestation (d 86 to parturition). ^a,b^ Means in the same row with uncommon superscripts differ (*p* < 0.05). ^c,d^ Means in the same row with uncommon superscripts differ (*p* < 0.10).

**Table 5:** Proximate and fatty acid composition of tissues from wethers born to ewes consuming endophyte-infected (E+) or endophyte-free (E−) fescue during MID (d 35-85) and LATE (d 86- parturition) gestation.

	Maternal Fescue Treatment ^1^				
	E−/E−	E+/E−	E−/E+	E+/E+	SE
Lambs (n)	11	10	10	11	
**Ground Lamb**					
Moisture, %	60.80	60.54	60.30	59.66	2.63
Crude protein, %	19.87	19.03	19.78	19.56	3.06
Total lipid, %	21.51	21.72	21.76	22.78	3.12
**Liver**					
Moisture, %	70.72	70.77	71.12	70.63	0.64
Crude protein, %	19.23	19.66	19.15	19.56	1.16
Total lipid, %	2.86	2.86	2.82	2.94	0.61
**Longissimus Muscle**					
Moisture, %	73.61	74.15	74.05	73.98	0.77
Crude protein, %	20.04	20.02	19.48	19.37	1.27
Total lipid, %	3.40	3.02	3.13	3.21	0.77
**Semitendinosus**					
Moisture, %	74.14	74.11	74.06	74.36	0.82
Crude protein, %	19.34	18.92	19.03	18.24	1.50
Total lipid, %	3.81	3.84	3.76	3.86	0.86

^1^ Fescue seed treatment: wethers born to ewes fed endophyte-free (E−) or endophyte-infected (E+) tall fescue seed during MID gestation (d35 to 85) and/or LATE gestation (d 86 to parturition).

**Table 6:** Fatty acid composition of tissues from wethers born to ewes consuming endophyte-infected (E+) or endophyte-free (E−) fescue during MID (d 35-85) and LATE (d 86- parturition) gestation.

	Maternal Fescue Treatment ^1^				
	E−/E−	E+/E−	E−/E+	E+/E+	SE
**Fatty Acids (Carbon number:double bond number, bond orientation-position), %**					
C14:0	1.63 ^b^	1.56 ^b^	1.81 ^a^	2.12 ^a^	1.09
C15:0	0.20 ^b^	0.19 ^b^	0.22 ^a^	0.25 ^a^	0.11
C16:0	20.07 ^d^	20.11 ^d^	20.49 ^c^	20.37 ^c^	1.28
C16:1 cis-9	1.60	1.56	1.64	1.62	0.32
C17:0	0.30 ^b^	0.31 ^b^	0.46 ^a^	0.33 ^b^	0.17
C18:0	18.72 ^b^	19.09 ^b^	19.02 ^b^	18.08 ^a^	1.22
C18:1 trans-9	0.23	0.19	0.21	0.16	0.32
C18:1 trans-10	7.27 ^a^	4.92 ^b^	5.27 ^b^	6.91 ^a^	1.99
C18:1 trans-11	0.06 ^d^	0.14 ^d^	0.27 ^c^	0.31 ^c^	0.67
C18:1 cis-9	33.56	35.44	34.39	32.99	3.05
C18:1 cis-11	1.86 ^c^	1.76 ^cd^	1.70 ^d^	1.80 ^cd^	0.35
C18:2 cis-9,12	5.90 ^a^	5.68 ^ab^	5.40 ^b^	5.79 ^a^	1.00
C18:3 cis-9,12,15	0.24	0.24	0.23	0.25	0.044
C20:2 cis-11,14	0.090	0.082	0.085	0.084	0.029
C20:3, cis-5,8,11,14	0.10	0.12	0.086	0.12	0.23
C22:5, cis-7,10,13,16,19	0.54	0.57	0.56	0.54	0.13
C22:6, cis-4,7,10,13,16,19	0.21	0.24	0.25	0.23	0.14
SFA	21.78 ^b^	21.74 ^b^	22.38 ^a^	22.57 ^a^	1.47
OCFA	0.50	0.50	0.68	0.58	0.24
MUFA	35.32 ^bc^	37.15 ^a^	36.21 ^ab^	34.79 ^c^	3.09
PUFA n-6 ^2^	9.28	9.27	8.91	9.19	1.36
PUFA n-3 ^3^	1.13	1.18	1.16	1.16	0.25
Ratio	10.84 ^a^	9.96^b^	9.92 ^b^	10.27 ^ab^	1.96

^1^ Fescue seed treatment: wethers born to ewes fed endophyte-free (E−) or endophyte-infected (E+) tall fescue seed during MID gestation (d35 to 85) and/or LATE gestation (d 86 to parturition). ^2^ Omega-6 Polyunsaturated fatty acids (PUFA, n-6). ^3^ Omega-3 Polynsaturated fatty acids (PUFA, n-3). ^a,b^ Means in the same row with uncommon superscripts differ (*p* < 0.05). ^c,d^ Means in the same row with uncommon superscripts differ (*p* < 0.10).

**Table 7:** Concentrations of plasma glucose and insulin, serum non-esterified fatty acids (NEFA) and revised quantitative insulin sensitivity chek index (RQUICKI) from wethers born to ewes consuming endophyte-infected (E+) or endophyte-free (E−) fescue during MID (d 35-85) and LATE (d 86- parturition) gestation at specific time points during postnatal growth.

	Postnatal Time Points				
	Birth	Wean	Grower	Finish	SE
**Glucose, mg/dL**					
E−/E−	57.51 ^b^	72.66 ^a^	72.29 ^a^	80.66 ^a^	6.78
E+/E+	55.13 ^b^	74.79 ^a^	76.96 ^a^	78.55 ^a^	5.87
**Insulin, ug/L**					
E−/E−	0.11 ^b^	0.25 ^ab^	0.39 ^a^	0.20 ^ab^	0.084
E+/E+	0.14 ^b^	0.23 ^ab^	0.38 ^a^	0.35 ^ab^	0.073
**Glucose:Insulin**					
E−/E−	510.4 ^a^	345.4 ^a^	218.0 ^b^	402.2 ^a^	68.31
E+/E+	428.6 ^a^	469.8 ^a^	237.9 ^b^	342.5 ^a^	59.16
**NEFA**					
E−/E−	-	0.91 ^a^	0.70 ^ab^	0.35 ^b^	0.200
E+/E+	-	0.72 ^a^	0.58 ^ab^	0.49 ^b^	0.158
**RQUICKI**					
E−/E−	-	0.99	1.15	2.93	1.27
E+/E+	-	3.25	0.90	1.30	1.10

^a,b^ Means in the same row with uncommon superscripts differ (*p* < 0.05).
